# DeepBeam: a machine learning framework for tuning the primary electron beam of the PRIMO Monte Carlo software

**DOI:** 10.1186/s13014-021-01847-w

**Published:** 2021-06-29

**Authors:** Zbisław Tabor, Damian Kabat, Michael P. R. Waligórski

**Affiliations:** 1 AGH University of Science and Technology, Al. Adama Mickiewicza 30, 30-059 Kraków, Poland; 2 Maria Sklodowska-Curie National Research Institute of Oncology Krakow Branch, Garncarska 11, 31-115 Kraków, Poland; 3 Cracow University of Technology, Podchorążych 1, 30-084 Kraków, Poland

**Keywords:** Machine learning, Deep learning, Monte Carlo, Beam simulation, Quality assurance (QA), Quality control (QC), Principal component analysis (PCA), Support vector regression

## Abstract

**Background:**

Any Monte Carlo simulation of dose delivery using medical accelerator-generated megavolt photon beams begins by simulating electrons of the primary electron beam interacting with a target. Because the electron beam characteristics of any single accelerator are unique and generally unknown, an appropriate model of an electron beam must be assumed before MC simulations can be run. The purpose of the present study is to develop a flexible framework with suitable regression models for estimating parameters of the model of primary electron beam in simulators of medical linear accelerators using real reference dose profiles measured in a water phantom.

**Methods:**

All simulations were run using PRIMO MC simulator. Two regression models for estimating the parameters of the simulated primary electron beam, both based on machine learning, were developed. The first model applies Principal Component Analysis to measured dose profiles in order to extract principal features of the shapes of the these profiles. The PCA-obtained features are then used by Support Vector Regressors to estimate the parameters of the model of the electron beam. The second model, based on deep learning, consists of a set of encoders processing measured dose profiles, followed by a sequence of fully connected layers acting together, which solve the regression problem of estimating values of the electron beam parameters directly from the measured dose profiles. Results of the regression are then used to reconstruct the dose profiles based on the PCA model. Agreement between the measured and reconstructed profiles can be further improved by an optimization procedure resulting in the final estimates of the parameters of the model of the primary electron beam. These final estimates are then used to determine dose profiles in MC simulations.

**Results:**

Analysed were a set of actually measured (real) dose profiles of 6 MV beams from a real Varian 2300 C/D accelerator, a set of simulated training profiles, and a separate set of simulated testing profiles, both generated for a range of parameters of the primary electron beam of the Varian 2300 C/D PRIMO simulator. Application of the two-stage procedure based on regression followed by reconstruction-based minimization of the difference between measured (real) and reconstructed profiles resulted in achieving consistent estimates of electron beam parameters and in a very good agreement between the measured and simulated photon beam profiles.

**Conclusions:**

The proposed framework is a readily applicable and customizable tool which may be applied in tuning virtual primary electron beams of Monte Carlo simulators of linear accelerators. The codes, training and test data, together with readout procedures, are freely available at the site: https://github.com/taborzbislaw/DeepBeam.

**Supplementary Information:**

The online version contains supplementary material available at 10.1186/s13014-021-01847-w.

## Introduction

External photon beam therapy (EBT) is nowadays the most common cancer radiotherapy modality. The key factors which determine the success of EBT are correct and accurate treatment therapy planning and quality assurance procedures prior to delivery of the therapeutic dose. Designing a therapy plan is a multi-dimensional optimization problem. The treatment planning system (TPS) designs therapy plan to match the therapy goals within specified clinical tolerances. Only after assuring that these goals are met, may the quality assurance procedures of dose delivery be implemented and the patient treated.

Currently, treatment planning systems calculate the dose distribution within an irradiated volume based on approximate algorithms like Pencil Beam Convolution (PBC) [1], Anisotropic Analytical Algorithm (AAA) [2] or ACUROSE XB (AXB) algorithm [3]. Monte Carlo (MC) modelling is an alternative approach to calculate dose distributions [4]. An advantage of MC over most analytical-based algorithms is that the latter are adequate for dose calculations in homogeneous media but they could be rather crude approximations whenever inhomogeneities are present. In contrast, MC simulations, while also based on approximations (mostly in the transport physics and cross sections estimation), have been demonstrated to be superior over analytical algorithms when it comes to dose distributions calculations in heterogeneous volumes [5]. However, while being potentially very accurate and extremely valuable in gaining thorough understanding of all phenomena related to dose deposition in various media, it is also a very challenging task [4]. This is due not only to the high computational effort required by MC modelling, but also because of the tuning process which must be carefully implemented to match MC-calculated doses and doses measured under controlled conditions. This tuning process involves finding an appropriate model of a primary electron beam of a medical linear accelerator being simulated.

Any MC simulation of dose delivery by megavolt photon beams from a medical linear accelerator commences by simulating the primary beam of high-energy electrons which leave the acceleration tube of the linac with an energy of several MeV impinging a tungsten target to generate megavolt photons via bremsstrahlung. The spatial distribution of dose delivered by the thus generated photon beam to a water phantom or to the patient’s tumour volume, crucially depends on the characteristics of the primary electron beam. Yet, the primary electron beam characteristics of any individual accelerator are unique. Moreover, the characteristics of their primary electron beams may vary not only between linacs of the same type, but may also vary in time within the same accelerator, due to ageing effects [6–8]. Unfortunately, the geometry and spectra of the primary electron beam are neither known exactly nor easily measurable, except by quite specialized equipment, which is not readily available in a typical clinical radiotherapy environment [9, 10].

For this reason, to run MC simulations, an appropriate model of the electron beam must be designed, which includes a model of its electron energy spectrum and model of its spatial distribution. Typically, no less than four parameters are needed to characterise a MC-simulated electron beam of a medical linear accelerator—namely the mean energy of electrons in the beam, the full width at half-maximum of the electron energy spectrum in the beam by assuming the energy distribution is a Gaussian function, the radial distribution of electrons in the beam, and the angular divergence of this beam. Clearly, in order to generate a clinically realistic MC simulation of dose delivery from any individual accelerator, the parameters of the model of the electron beam must be determined specifically for that accelerator [11].

For above-discussed reasons, availability of a well-defined and realistically executable procedure of specifying the parameters of the model of a primary electron beam in an individual linear accelerator is of major importance in the subsequent application of MC-based modelling of clinical procedures using this particular accelerator. Notably, parameters of a MC model of the electron beam of a linac can only be determined indirectly by analysing a number of depth and lateral dose-profiles measured in specified conditions, best in a standard water phantom.

Due to the fundamental importance of this issue, several studies have been published proposing various experimental setups and methods of such analysis [6–9, 11–17]. These studies are briefly reviewed in Related works section of Additional file 1 accompanying this paper. It follows that in most cases a trial-and-error approach has been adopted to determine parameters of a model of a primary electron beam. No phenomenological model enabling the values of primary electron beam parameters to be estimated directly from the measured dose profiles has been proposed. The proposed tuning procedures are often very specific with respect to the data required to determine the parameters of a model of a primary electron beam.

In contrast to such studies, we propose a flexible framework, termed DeepBeam, such that its user may collect the profile data using dosimetry tools and protocols at her/his disposal and select the profiles to be measured according to her/his best experience. Then, a regression model is created which can be directly and routinely used to estimate the parameters of the model of the primary electron beam. The proposed framework, apart from tuning electron beams of Monte Carlo simulators of real linear accelerators may also be applied in routine quality assurance of an operating linear accelerator—not only to verify its beam stability via dose profile analysis, but also to indicate which of the beam parameters had likely changed and by how much. The complete code of this framework and the data used for training the regression models are freely available at https://github.com/taborzbislaw/DeepBeam.

## Material and methods

### The MC simulator and sources of data

While the proposed framework for tuning primary electron beams of MC simulators of linear accelerators can be used for any such simulator, the present study is based on data generated by the PRIMO simulator, version 0.1.5.1307 [18] (www.primoproject.net). PRIMO is a freely-distributed application used for simulating radiation transport during radiotherapy [8, 17, 18]. It is based on the PENELOPE 2011 [19] general purpose Monte Carlo engine and allows simulation of dose delivery to be performed for a few linear accelerator models, based on their geometry, as provided by their manufacturers. This last feature is especially important, as details of accelerator geometry are usually confidential and may not be available from the manufacturer, even upon request. Hence, using PRIMO, attention could be focussed on the primary goal of designing a framework for tuning the electron beam of this simulator without undue concern with simulation details related to the physics, materials or the geometry configuration of the simulated accelerator system.

All simulations were run using the PRIMO Varian Clinac 2300 C/D simulator operating in photon mode at a nominal energy of 6 MV. Electron beam simulation in PRIMO is configured by specifying values of four beam parameters: *E*—the initial electron beam energy (in MeV), σ_*E*_—the full-width-at-half-maximum (FWHM) of the primary beam energy distribution (in MeV), *s*—the focal spot FWHM (in cm), and α—the angular beam divergence (in degrees). The developed framework should however be readily adaptable if different primary beam parameters were specified in the PRIMO simulator, or if other MC simulators of linear accelerators were applied.

#### Simulated input data

To generate training data for the machine learning framework, the simulations were run for a total of 300 tuples (*E*, σ_*E*_, s, α) within the set *S* such that:

1 $$\begin{array}{c}S=\{\left(E,{\sigma }_{E},s,\alpha \right):E\in \{\mathrm{5.6,5.8,6.0,6.2,6.4}\},{\sigma }_{E}\in \{\mathrm{0.0,0.5,1.0}\},\\ s\in \{\mathrm{0.0,0.1,0.2,0.3,0.4}\},\alpha \in \{\mathrm{0,1},\mathrm{2,3}\}\}.\end{array}$$

At the first simulation stage, 10^8^ histories (a history corresponds to a single electron of the virtual primary beam) were simulated for each tuple (*E*, σ_*E*_, s, α) and the phase-space file (PSF) above the secondary collimators was saved for further purposes. At this first stage, the splitting roulette variance reduction technique [20] was used with the size of the splitting region set to the largest region, i.e. to the 40 × 40 cm^2^ field. The saved PSFs were then used to simulate radiation transport to a homogeneous cubic water phantom for three fields: 3 × 3 cm^2^, 10 × 10 cm^2^, and 30 × 30 cm^2^. The size of the phantom was set to 50 × 50 × 50 cm^3^. The doses in the phantom were tallied within a regular grid of 0.5 × 0.5 × 0.5 cm^3^ voxels. The respective faces of the phantom were set parallel to the respective main axes of the coordinate frame of reference of the accelerator. The main axis of the phantom coincided with the photon beam axis. The source-to-surface distance (SSD) was set at 100 cm, the isocentre being located at the front surface of the phantom. Splitting in the water phantom was selected as the variance reduction method [20] at this simulation stage, with a splitting factor of 300. The uncertainty of the dose values tallied in the water phantom always remained within 1.5% (which corresponds to two standard deviations of MC calculated dose). The calculated 3D spatial distribution of doses within the phantom was saved to a text file, separately for each tuple (*E*, σ_*E*_, s, α) and for each field. A total of 900 3D dose files were collected. Each 3D dose file contained 10^6^ dose values calculated by PRIMO at (*x*,*y*,*z*) coordinates given by the following coordinate ranges:

2 $$\begin{array}{c}x\in \{-25+0.25+0.5*i,i=1...100\}\\ y\in \{-25+0.25+0.5*j,j=1...100\}\\ z\in \left\{0.25+0.5*k,k=1...100\right\},\end{array}$$

where the *z* axis is parallel to the radiation field axis. To generate testing data for the machine learning framework, the simulations were run further for 25 tuples (*E*, σ_*E*_, s, α) with primary beam parameters sampled randomly from the following sets of values:

3 $$\begin{array}{c}E\in \{5.65+i\cdot 0.05,i=0...14\}\setminus \{\mathrm{5.8,6.0,6.2}\}\\ {\sigma }_{E}\in \{0.1+i\cdot 0.1,i=0...8\}\setminus \{0.5\}\\ s\in \{0.05+i*0.1,i=0...3\}\\ \alpha \in \{0.5+i*0.25,i=0...9\}\setminus \{\mathrm{1,2}\}\end{array}$$

Applying the above sampling scheme, it was assured that the primary electron beam parameters (*E*, σ_*E*_, s, α) in the testing set never coincided with parameters used for generating the training set, and consequently, that the electron beam parameters for the testing set were well separated from the electron beam parameter selected for training.

All simulations were run using the PlGrid infrastructure (Prometheus grid, https://kdm.cyfronet.pl/portal/Main_page) and required a total real time of about 2.5 months. During the simulation period 12 Prometheus nodes run the PRIMO software, each node equipped with two Intel Xeon E5-2680v3 processors, 24 cores in total, and 128 GB RAM. The simulation of a single case, i.e., of three fields for a single tuple (*E*, σ_*E*_, s, α), required about 40 CPU hours. As the operating system installed on the nodes is Linux CentOS 7, while PRIMO is a Windows application, *wine* software (https://www.winehq.org/) was installed and configured in order to use PRIMO in graphic mode under Linux exactly as if Windows were the operating system.

#### Measured input data

Dose profiles were measured in water for the 6 MV photon beam of a clinically exploited Clinac 2300C/D medical accelerator at the Krakow Branch of the National Research Institute of Oncology. A PTW MP3 Water Phantom and PTW Markus Type 23,343 and PTW Semiflex Type 31,010 ionization chambers were used for dosimetry. PTW Mephysto software was applied for data collection. Three experimental setups of dose profile measurements were arranged, as described in more detail in the Results section.

### Applied models and computational framework

The task to solve is a regression problem, i.e., given dose profiles in a water phantom, the parameters (*E*, σ_*E*_, s, α) of the primary electron beam are to be estimated. To prepare training and test data, each 3D dose spatial distribution was normalized to the dose value calculated along the photon beam axis at the depth of maximum (D_max_ = 1.4 cm), which was then set to 100% (such normalization is not essential if not implemented in a clinical measurement system). Next, from each 3D dose file six profiles were extracted: one depth profile along the axis of the radiation field, and five lateral profiles at depths: 1.4 cm, 5 cm, 10 cm, 20 cm, and 30 cm. To match the resolution of the simulated profiles and the typical spatial resolution of clinical dosimetry systems (usually 1 mm), linear interpolation was applied to the tallied simulated doses during profile extraction. Additionally, as PRIMO assumes the electron beam spot to be of circular shape, the lateral dose profiles extracted from the 3D dose files consisted of averages over two perpendicular lateral dose profiles over the *x* and *y* directions. Such averaging is not a necessary condition and may be skipped if a more complex, e.g., elliptic, electron spot shape is assumed by the accelerator simulator.

The extracted dose profiles (18 profiles for each tuple (*E*, σ_*E*_, s, α)) represent a reasonable maximum set Prof_*MAX*_ of dose profiles to be used in the proposed machine learning framework. Moreover, the extracted depth dose profiles span the range of z ∈  < 0.3 cm, 49.7 cm > , while all the extracted lateral dose profiles span the range of x ∈  < -24.7 cm, 24.7 cm > , i.e., the maximum ranges for the geometry of the simulated water phantom and for the spatial resolution of the grid of tallied dose values.

The proposed framework is customizable, meaning that any subset of the dose profiles can be selected from the complete set of dose profiles to match the needs of an individual user. The ranges over which the profiles are measured can also be arbitrarily selected to match the measurement ranges of real profiles. For example, the user may decide to build her/his regression model which predicts the parameters of the model of the electron beam (*E*, σ_*E*_, s, α) from the depth dose profiles and from lateral dose profiles at 10 cm depth, all collected for 10 × 10 cm^2^ and 30 × 30 cm^2^ fields, depth dose profiles measured up to 35 cm, and lateral dose profiles measured over the ranges between − 10 cm and + 10 cm and between -20 cm to + 20 cm, for 10 × 10 cm^2^ and 30 × 30 cm^2^ fields, respectively. Given these user-defined constraints the framework finds the optimum regression model, as described in the following sections.

#### PCA + SVR regression model

Let Prof = {Prof_1_, Prof_2_,…, Prof_n_} represent a user-selected subset of Prof_MAX_. A user-selected spatial range *Range*_*i*_ is associated with each Prof_i_ (*Range*_*i*_ would typically be the user-dependent spatial range over which Prof_i_ is measured under clinical settings). Each subscript *i* corresponds to a unique field size and a unique dose profile type (either depth or lateral, at one of the five depths: D_max_ = 1.4 cm, 5 cm, 10 cm, 20 cm, or 30 cm).

As each dose profile Prof_i_ is sampled within a given spatial resolution (usually 1 mm), it may be considered a 1D vector of some dimensionality (dependent of the sampling resolution and the sampling range *Range*_*i*_). The regression task which is to be solved can be formulated as follows:

4 $$Par={f}_{Par}\left({Prof}_{1},{Prof}_{2},...,{Prof}_{n}\right)+{\epsilon }_{Par},$$

where *Par* is any element of the tuple (*E*, σ_*E*_, s, α), *f*_*Par*_ is the regression function and ε_*Par*_ is the residual term. The components of each Prof_i_ are however strongly correlated as they represent dose values measured at neighbouring spatial locations. For this reason, the model given by Eq. (4) may not be very effective, as the set of explanatory variables (arguments of *f*_*Par*_) contains a high contribution of redundant information.

To resolve this redundancy problem dimensionality reduction is applied. Typically, dose profiles are specified by applying some ad-hoc features, such as width at half maximum, width of penumbra regions, “wing heights” in lateral profiles, etc. Here, rather than rely on such hand-crafted features, Principal Component Analysis (PCA) is applied to the analysed profiles [21]. PCA is a method for finding uncorrelated variables from correlated ones (in our case—consecutive dose values along depth and lateral profiles). Finding such new variables reduces to solving an eigenvalue/eigenvector problem, and the new variables are linear combination of the old ones. Moreover, each PCA feature is assigned a percentage of the total variance of profile shapes it explains. As demonstrated in what follows, three most important PCA features usually explain over 98% of the variability of shapes of the training dose profiles. PCA reduces the dimensionality of the space of explanatory variables by a factor of 10^2^—the final set of features consists of 3*n* elements (explanatory variables)—three features for each profile Prof_i_ in Prof. The learnt PCA models were saved in respective files (a separate PCA model *M*_*PCA,i*_ file for each index *i*) and subsequently used at the stage of model testing.

Clearly, for each *i—*index there are 300 training profiles {Prof_*i,*1_, Prof_*i,2*_*,…*,Prof_*i,*300_} corresponding to 300 different tuples {(*E*, σ_*E*_, s, α)_1_, (*E*, σ_*E*_, s, α)_2_,…, (*E*, σ_*E*_, s, α)_300_} and a single PCA model *M*_*PCA,i*_ which extracts three features (*F*_*i,1*_, *F*_*i,2*_, *F*_*i,3*_*)*_*k*_ from Prof_i*,k*_. Hence, the regression problem, after dimensionality reduction, becomes:

5 $$Par={f}_{PAR}^{PCA}\left({F}_{\mathrm{1,1}},{F}_{\mathrm{1,2}},{F}_{\mathrm{1,3}},{F}_{\mathrm{2,1}},{F}_{\mathrm{2,2}},{F}_{\mathrm{2,3}},...,{F}_{n,1},{F}_{n,2},{F}_{n,3}\right)+{\epsilon }_{PCA},$$

where *Par* is any element of the tuple (*E*, σ_*E*_, s, α), ${f}_{PAR}^{PCA}$ is the PCA-based regression function and ε_*PCA*_ is the residual term. To learn the regression functions, the following training set, *Tr*, was applied:

6 $$Tr=\left\{{\left(E,{\sigma }_{E},s,\alpha \right)}_{K},\left\{{\left({F}_{\mathrm{1,1}},{F}_{\mathrm{1,2}},{F}_{\mathrm{1,3}}\right)}_{K},{\left({F}_{\mathrm{2,1}},{F}_{\mathrm{2,2}},{F}_{\mathrm{2,3}}\right)}_{K},...,{\left({F}_{n,1},{F}_{n,2},{F}_{n,3}\right)}_{K}\right\},K=1..300\right\}.$$

Support Vector Regression (SVR) with radial basis function (rbf) kernel was selected as the regressor [22] though other options are also available. SVR with rbf kernel first transforms the explanatory variables (PCA features in our case) to some high dimensional space and then, in this high dimensional space, replaces nonlinear relationship of Eq. (5) with a multilinear one between transformed explanatory variables and explained variables (electron beam parameters in our case). This high dimensional multilinear relationship is then used to make predictions. The best regression models were selected using a fivefold cross validation run on *Tr*. After training, four ${f}_{PAR}^{PCA}$ regressors were obtained, one per *E*, σ_*E*_, s, and α. The regression models were saved to files and subsequently used in testing.

#### The deep learning regression model

**Fig. 1:**
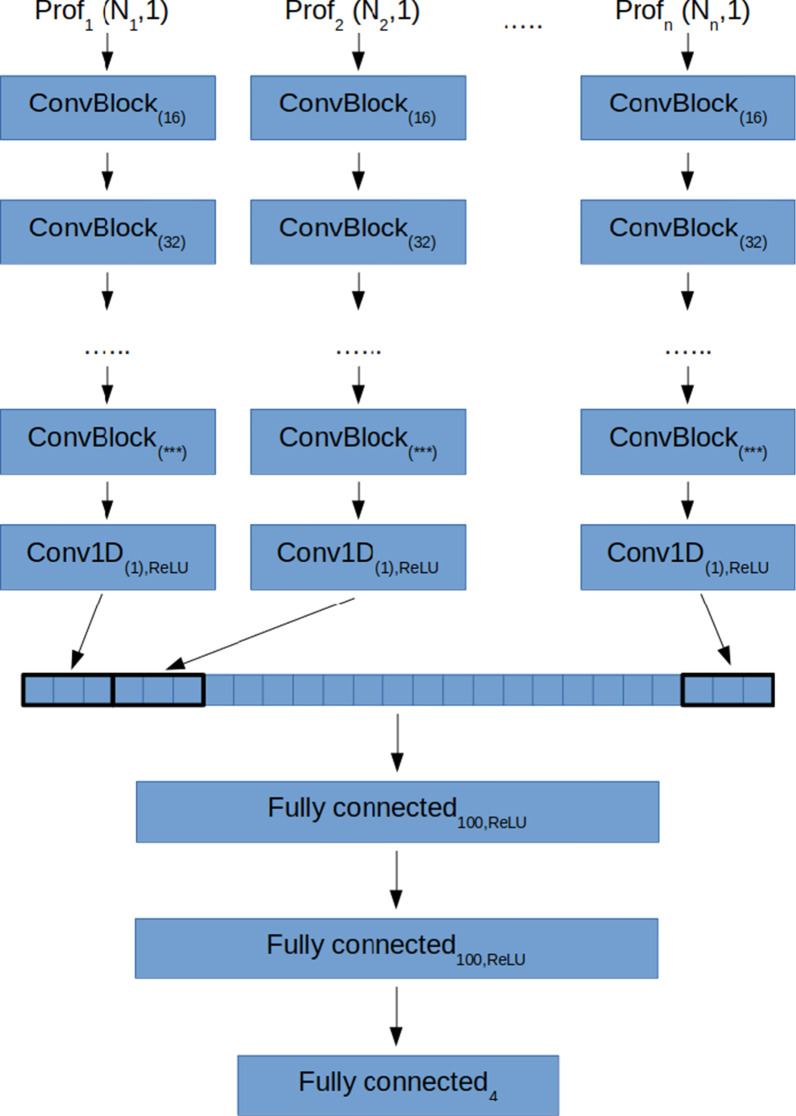
Scheme of the deep learning regression model architecture

The processing pipeline described in the previous section consists of two separate steps: feature extraction, and training of four regressors. In the current section an end-to-end regression model is described which, during training, learns both dose profile data representation and regression functions simultaneously for all primary beam parameters (*E*, σ_*E*_, s, α). The model presented here is based on deep learning. The architecture of the deep learning (DL) model is outlined in Fig. 1.

The architecture is designed to follow the same processing steps as the approach described in the previous section. In short, each Prof_i_ in Prof is a separate input for the DL model and is processed by a separate encoder block. Each encoder block consists of a few convolution blocks. Each convolution block consists of two 1D convolutions (filter size equal to 3, number of filters equal to 16, 32, 64, etc. in the consecutive convolution blocks, ReLU activation) followed by a MaxPool1D layer which reduces the size of the data by a factor of two. The number *L* of convolution blocks in each encoder block is selected based on the length *N* of the input of this block, according to the formula *L* = int(log_2_*N*/3), i.e., the number of features learnt by any encoder block cannot be less than 3. Each encoder block ends with 1D convolution with a single filter of unit size. The outputs of the encoder blocks are then concatenated to form a 1D vector of features (in analogy to PCA features). This feature vector is then processed by two fully connected layers of size 100 and ReLU activation. The output of the last fully connected layer is next fed into the final fully connected layer with four outputs and no activation. These outputs are expected to deliver estimates of *E*, σ_*E*_, s, and α.

The training data *Tr*_*DL*_ for the DL model is:

7 $${Tr}_{DL}=\left\{{\left(E,{\sigma }_{E},s,\alpha \right)}_{K},\{{Prof}_{1,K},{Prof}_{2,K},...,{Prof}_{n,K}\},K=1...300\right\}.$$

The model is trained for 300 epochs using the Adam optimizer and a constant learning rate equal to 0.0001. The loss function selected for this regression problem was mean square error between the model outputs and the values of primary electron beam parameters for which the dose profiles at the model input were obtained. A 20% portion of the training set was randomly selected for model validation. The best model found during training was saved to a file and used in subsequent testing.

#### Testing the models

At the stage of model testing, the testing profiles were fed at the input of either the PCA + SVR or DL models. The PCA + SVR model first extracts the features from the testing profiles based on PCA models learnt on the training set. These test features are then processed by SVR regressors which return the predicted values of *E*, σ_*E*_, s, and α. In the case of the DL model the raw testing profiles are fed at the input of the DL model which returns the predicted values of *E*, σ_*E*_, s, and α. The true and predicted values of *E*, σ_*E*_, s, and α are then compared using correlation analysis and linear regression.

#### Optimizing the solution with profiles reconstructed from regression results

**Fig. 2:**
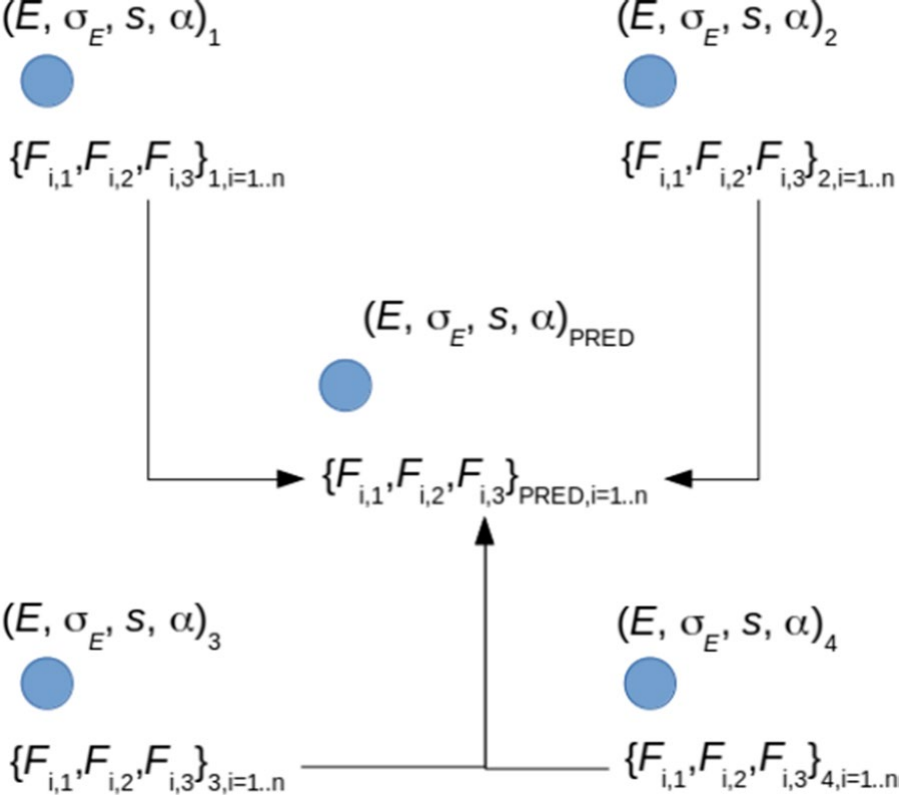
Scheme of the method of reconstructing profiles from regression results

The regression results can be further improved by minimizing the difference between the actual profiles being fed at the input of regressors and profiles reconstructed from the regression results. In particular, the parameters of the model of the primary electron beam for the training set *Tr* (Eq. (6)) have the form of a regular grid *S*, defined in Eq. (1), embedded within a 4D hypercube *H*. With every node *Q* of *S* associated are PCA features corresponding to the dose profiles determined for primary electron beam model parameters (*E*, σ_*E*_, s, α)_Q_ assigned to *Q*. The regressions return a point *P* = (*E*, σ_*E*_, s, α)_PRED_ within *H* (see Fig. 2 for a 2D example). Consecutively, using interpolation, PCA features corresponding to *P* may be determined, and next an inverse PCA transform applied to them in order to reconstruct profiles from the results of regression models (*E*, σ_*E*_, s, α)_PRED_. Thus, for each Prof_i_ in Prof a reconstructed profile RecProf_i_(*P*) obtains, which in general differs from Prof_i_. This difference can then be further minimized using one of several optimization methods, with (*E*, σ_*E*_, s, α)_PRED_ as the starting point for such minimization. Namely, beginning with *P* = (*E*, σ_*E*_, s, α)_PRED_, *P*_*MIN*_ = (*E*, σ_*E*_, s, α)_MIN_ is sought, such that:

8 $${P}_{MIN}=\underset{P\in H}{argmin}{\sum }_{i=1}^{n}{w}_{i}{\|{Prof}_{i}-{RecProf}_{i}\left(P\right)\|}^{2},$$

where *w*_*i*_ is the weight assigned to the *i*-th profile. In the experiments all *w*_*i*_ were set to unity but in general the user may set these according to her/his actual needs. The minimization problem defined in Eq. (8) was solved using the SLSQP method [23].

#### Applied software

All models were implemented in Python 3.6.10. The *scipy* library (version 1.5.2) was used to implement regression models using PCA and SVR. The same library was used to run interpolation over 3D dose distributions to extract dose profiles, optimization of the regression results and profile reconstruction from regression results. The DL model was implemented using the *keras* (version 2.3.1) library. All codes, pretrained models, as well as training and testing data, are freely available at https://github.com/taborzbislaw/DeepBeam.

## Results

### Analysis of simulated training data

Detailed analysis of simulated training data formed the basis for model design decisions and aided in selecting the best hyperparameters (that is, parameters C and epsilon of SVR, see for example https://scikit-learn.org/stable/modules/generated/sklearn.svm.SVR.html) for the developed model. Following these decisions, a final verification of the performance of the model was performed using only test data. Thus, test data were never used in model fine-tuning.

The first issue considered was what number of PCA features extracted from the profiles explains what fraction of the variability in the shapes of profiles. The results shown in Selecting the number of PCA features section of Additional file 1 indicate that three PCA features suffice in explaining most of the variability of the shapes of profiles.

The second issue considered was how selection of dose profiles influences precision of estimation of the parameters of a primary electron beam model. The results shown in Selecting dose profiles section of Additional file 1 indicate that a total of six profiles—one depth profile and two lateral profile, and any two of three fields (3 × 3 cm^2^, 10 × 10 cm^2^, or 30 × 30 cm^2^) would be sufficient in obtaining precise predictions of *E*, s, and α values.

Considering the above-discussed results obtained using training, the final design decision was made to train regressors based on PCA features extracted from a total of six profiles, i.e., three profiles (depth, lateral at D_max_ = 1.4 cm depth and lateral at 10 cm depth) of two field sizes (10 × 10 cm^2^ and 30 × 30 cm^2^).

**Fig. 3:**
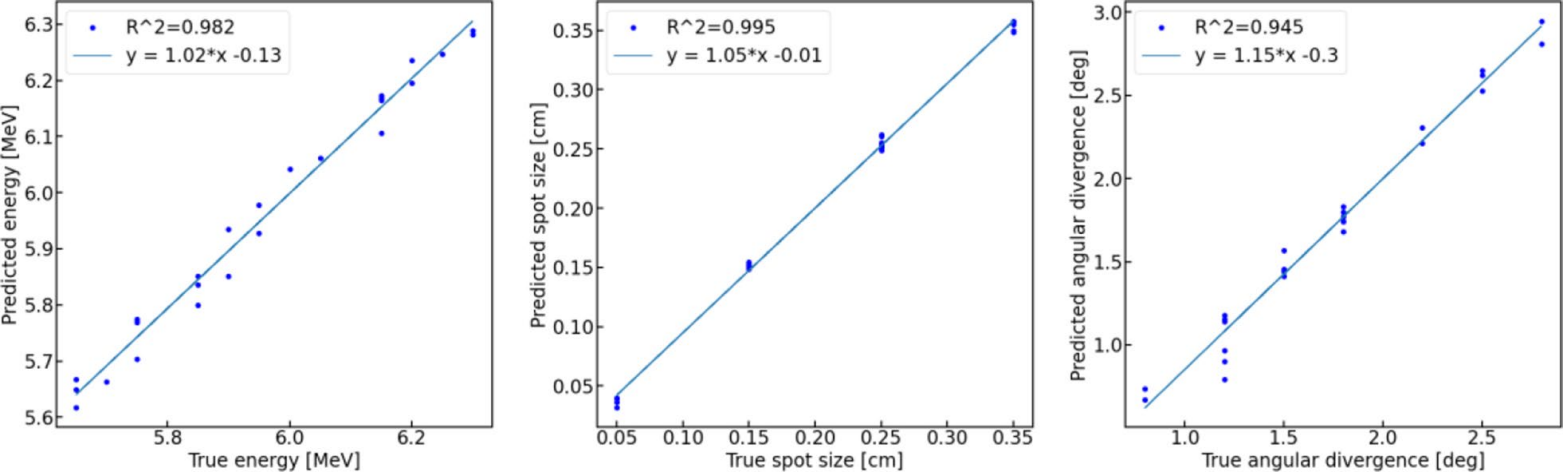
Testing results for the PCA + SVR model

The testing results for the PCA + SVR model are shown in Fig. 3. Models trained on the training data were applied to previously unseen testing data and the predicted values of primary beam parameters compared to the ground truth data, i.e., the values of primary beam parameters applied in the generation of simulated profiles. Notably, the values of the coefficient of determination for the testing data were only slightly lower than those obtained for the training data—implying that the regressors were not overfitted. Also shown in this figure are best-fitted linear regression lines to demonstrate the precision with which the model is able to predict the primary beam parameters. The slopes of these regression lines are all close to 1.0. The prediction errors were estimated as values of standard deviation of the difference between the true and predicted values of the primary electron beam parameter, and were equal to 0.03 MeV, 0.007 cm, and 0.13 degrees for *E*, s, and α, respectively.

**Fig. 4:**
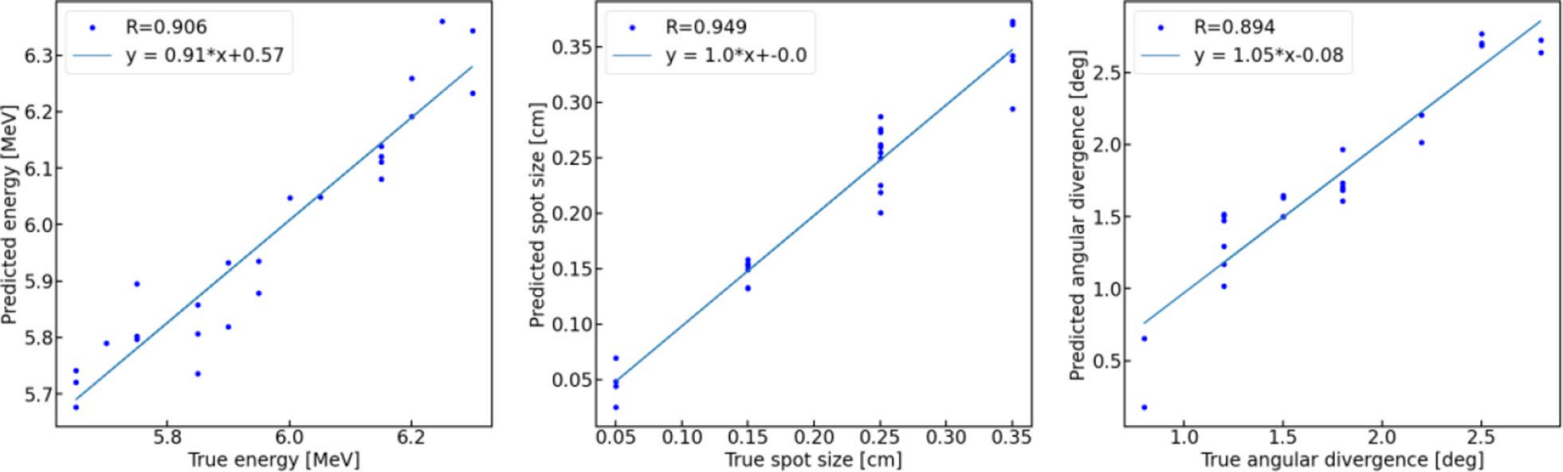
Testing results for the deep learning (DL) model

The testing results for the deep learning model trained on the same set of profiles as those used for the PCA + SVR model are shown in Fig. 4. The results for the deep learning model are slightly inferior to those obtained for the PCA + SVR model which is not surprising, since the deep model was trained only on 300 sets of profiles, which may not be sufficient for a deep learning task. Further refinements would certainly be possible, however in that case more data would need to be generated. Yet, as demonstrated in the next section, both models offer good starting points for optimization-based estimates of the parameters of a model of the primary electron beam from clinical measurements, leading to virtually the same final results. The prediction errors for the deep model were equal to 0.065 MeV, 0.023 cm, and 0.21 degrees for *E*, s, and α, respectively.

Discrepancies between the slopes of the best fit lines and the ideal 1.0 value are due to the noise present in the training data, which, although being relatively low (1.5%) is however higher than that in real measurements. Decreasing the noise level to 0.5% would however increase the computation time by a factor of 9 which is unrealistic in view of the computational expense. The optimization procedure which follows the regression, as described in the previous section, resolves this issue.

### Analysis of clinical data

**Fig. 5:**
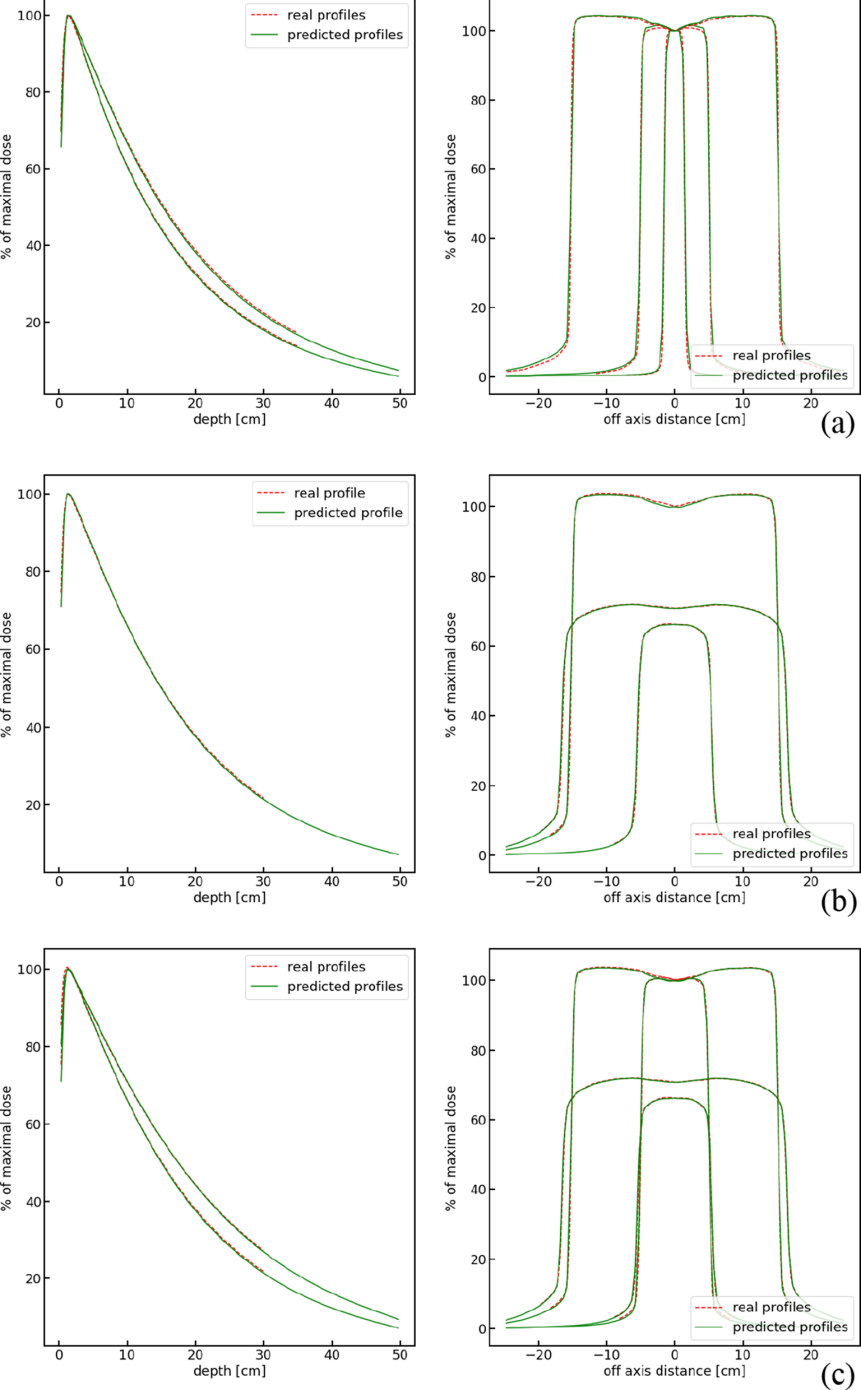
Measured and reconstructed dose profiles. Measured (real) depth and lateral profiles and respective profiles reconstructed using PCA and the finally estimated beam parameters (listed in column 6 of Table 1), for three cases of experimental design. The specification of profiles compared in each panel are listed in columns 2 and 3 of Table 1: **a** Case 1; **b** Case 2; **c** Case 3

The developed framework was used to find the values of primary electron beam parameters which could best reproduce real profiles measured using the 6 MV photon beam of a Clinac 2300C/D medical accelerator in a PTW MP3 Water Phantom. The applied input fields and profiles, obtained beam parameters, and mean errors of the reconstructed dose distributions against those measured, for three cases of experimental setups discussed below, are gathered in Table 1. The measured and reconstructed profiles for the respective sets of input profiles in each of these three cases are compared in Fig. 5.

**Table 1:** Results of SVR regression and deep model analysis of clinical profile data

Case ID	Applied Fields	Applied Profiles	Beam parameters			Mean absolute error between measured and reconstructed profiles [%]	1D Gamma passing rate between measured and reconstructed profiles [%]
			SVR regression E_PRED_, s_PRED_, a_PRED_	DL regression E_PRED_, s_PRED_, a_PRED_	Final estimation E_FINAL_, s_FINAL_, a_FINAL_		
1	3 × 3 cm^2^	Depth profile	5.54 MeV, 0.0 cm, 1.97^O^	6.02 MeV, 0.0 cm, 2.35^O^	5.86 MeV, 0.0 cm, 2.44^O^	0.33	100.0
		Lateral at Dmax				0.94	92.9
	10 × 10 cm^2^	Depth profile				0.50	100.0
		Lateral at Dmax				1.03	95.7
	30 × 30 cm^2^	Lateral at Dmax				0.90	97.6
2	10 × 10 cm^2^	Depth profile	5.47 MeV, 0.23 cm, 2.08^O^	5.50 MeV, 0.18 cm, 2.41^O^	5.60 MeV, 0.25 cm, 2.39^O^	0.33	100.0
		Lateral at 10 cm depth				0.41	100.0
	30 × 30 cm^2^	Lateral at Dmax				0.49	98.6
		Lateral at 10 cm depth				0.26	100.0
3	10 × 10 cm^2^	Depth profile	5.43 MeV, 0.24 cm, 1.95^O^	5.62 MeV, 0.27 cm, 2.47^O^	5.60 MeV, 0.24 cm, 2.41^O^	0.35	100.0
		Lateral at Dmax				0.75	96.3
		Lateral at 10 cm depth				0.40	99.4
	30 × 30 cm^2^	Depth profile				0.26	100.0
		Lateral at Dmax				0.43	98.1
		Lateral at 10 cm depth				0.20	100.0

The results for three experimental setups (Cases 1–3)) are reported in the table. The applied input fields and profiles are shown in columns 2 and 3. Values of PCA + SVR or DL- predicted beam parameters are shown in in columns 4, 5, and 6. The mean absolute differences between the measured profiles and profiles reconstructed using final estimation parameters, and the corresponding 1D gamma index passing rates for dose tolerance equal to 3% of dose at depth of maximum absorption and distance to agreement tolerance equal to 3 mm, are shown in columns 7 and 8

Three experimental design cases were investigated, where different sets of measured profiles (as shown in the second and third columns of Table 1) were used as input. In each of the three cases, the models were trained on a set of training profiles corresponding to those measured, after suitable adjustment of the ranges of the training profiles. Following this training, the measured profiles were then input to the trained PCA + SVR or DL models to obtain the values of the parameters of the model of the primary electron beam, *E*_*PRED*_, *s*_*PRED*_, and α_*PRED*_, shown in the fourth and fifth columns of Table 1, respectively. Because there was no possibility to train a regressor for predicting the value of σ_*E*_, σ_*E*_ = 0.50 MeV was consistently used throughout. These initial predictions were next fed as input to the reconstruction-based minimization procedure. After optimizing these predicted values for either model, usually identical (or very similar) sets of finally estimated parameter values: *E*_*FINAL*_, *s*_*FINAL*_, and α_*FINAL*_, shown in column 6 of Table 1, were obtained. These finally estimated electron beam model parameters values were then used to calculate the reconstructed dose profiles. The mean values of absolute differences between the measured and reconstructed profiles, and the corresponding 1D gamma index passing rates for dose tolerance equal to 3% of dose at depth of maximum and distance to agreement tolerance equal to 3 mm, are given in the last two columns of Table 1. The measured and reconstructed profiles in each of the three experimental cases are compared in Fig. 5.

The first set of measured profiles *(Case 1)* consisted of five profiles: two depth profiles and three lateral profiles, one of which was measured at the depth of D_max_ = 1.4 cm, as listed in Table 1. The depth profiles were measured to a depth of 35 cm while the ranges of measurement of lateral profiles were adjusted to the field size. The ranges of training data for this set of profiles were adjusted to the ranges of real measurements prior to being applied to train the PCA + SVR or DL models. The measured and reconstructed profiles for this case are shown in Fig. 5a.

The second set of measurement profiles *(Case 2)* consisted of one depth profile and three lateral profiles, one of which was measured at depth D_max_, listed in column 2 of Table 1. The depth profile was measured to a depth of 30 cm while the ranges of measurement of lateral profiles were adjusted to the field size. The measured and reconstructed profiles for this case are shown in Fig. 5b.

Finally, the third set of measurement profiles *(Case 3)* consisted of six profiles: a depth profile and two lateral profiles, both at D_max_ depth. The depth profile was measured to a depth of 30 cm while the ranges of measurement of lateral profiles were adjusted to the field size. The measured and reconstructed profiles for this case are shown in Fig. 5c.

Commenting generally on the results obtained, one should note the remarkably consistent estimates of beam parameters obtained using either the PCA + SVR or the deep learning models, and the excellent agreement between the reconstructed and measured profiles, especially using the set of measured profiles and fields in the Case 3 study. However, even in the Case 1 study, the somewhat higher discrepancies observed at the borders of the lateral fields are to be expected. Over such regions of high dose gradients, higher uncertainties may be due to measurement uncertainties, to averaging of input data by the phantom software or to averaging of data in the training profiles, all affecting the quality of reconstructed profiles over such regions. The excellent agreement between the reconstructed and real profiles is confirmed by the low values of mean absolute errors displayed in column 7 of Table 1 in most cases ranging around 0.5% and exceeding 1% only once, and by high gamma passing rates.

**Fig. 6:**
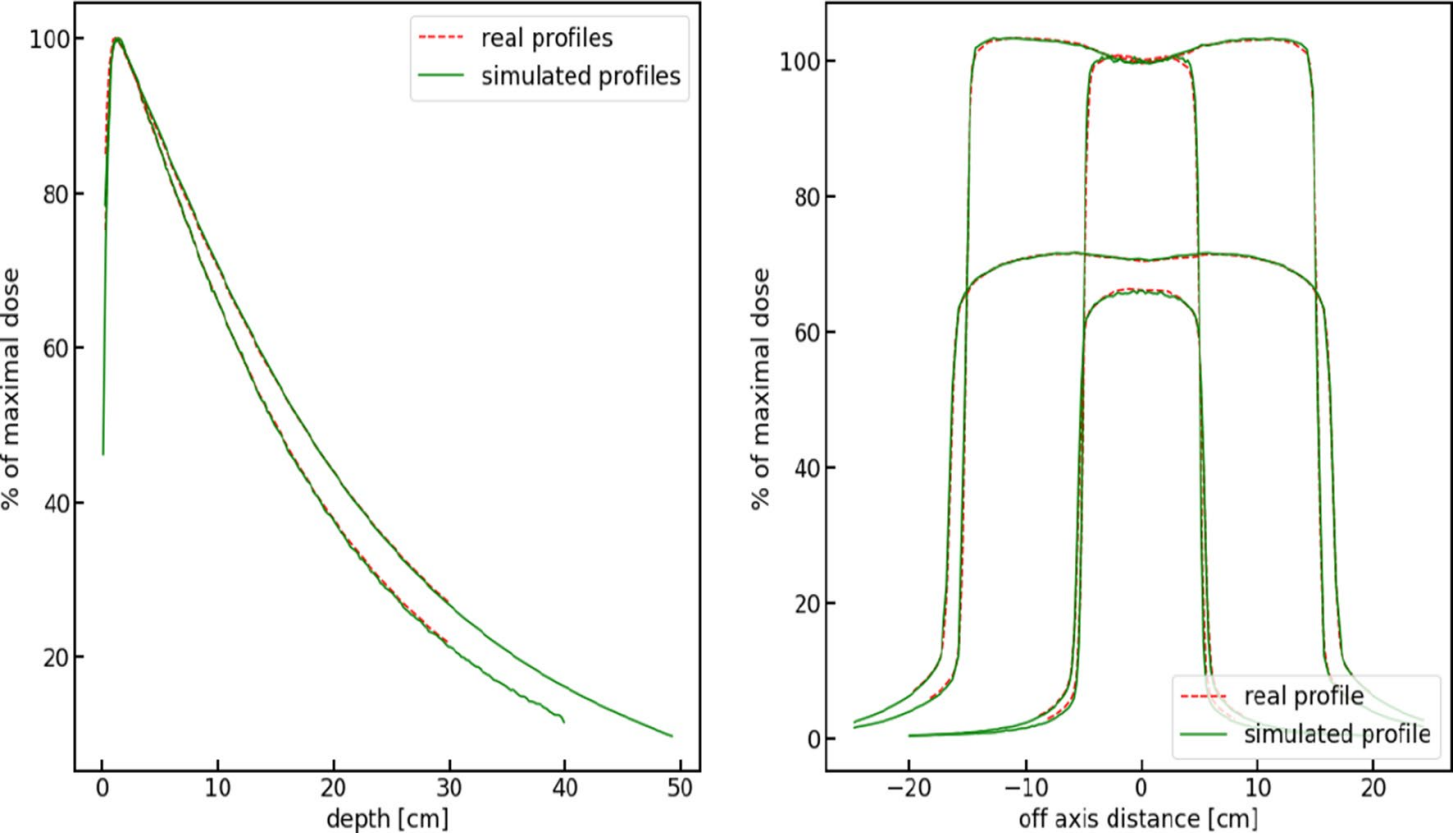
Measured and simulated dose profiles. Measured (real) depth and lateral profiles and respective simulated profiles. The simulation was run for 10^9^ histories for experimental settings and virtual primary electron beam profile corresponding to Case 3

While there is a very good agreement between measured and profiles reconstructed from regression results, based on PCA models, a final check of the proposed framework and must be comparison of measured and simulated profiles. To this end, the dose delivery was simulated for 10^9^ histories, using the virtual primary electron settings determined for Case 3 measurement experiment. Real profiles and simulated profiles for this case are compared in Fig. 6.

## Discussion

Development of a method to relate specific features of the in-phantom measured dose distributions with values of the parameters of the model of the electron beam in a medical accelerator was the prime motivation in this work. Ideally, for the solution of such a task to be of practical utility, it should be delivered as a model which accepts at its input a well-defined assembly of measured profiles, returning estimated values of the parameters characterizing the primary electron beam of a given accelerator, together with dose profiles reconstructed based on these estimated parameters. Availability of an elsewhere-developed complete MC model of the accelerator, i.e., of the PRIMO Monte Carlo software, and development and successful application of statistical learning technology made it possible to accomplish this task.

The solution presented in this study is flexible and readily usable. Based on Monte Carlo simulation data, a set of models was developed which extract features from a user-defined collection of dose profiles to estimate primary electron beam model parameters from such features and returns reconstructed profiles for comparison with those measured and used as input. In contrast to all the work published so far, the characteristics of the dose profile shapes and the regression functions are both machine-learned and collected in a data-dependent manner. Neither hand-crafted shape features nor ad-hoc regression functions need to be applied, these being replaced by a well-established background of statistical learning. The two models developed in this work—one based on PCA feature extraction and SVR regression and another, based on end-to-end deep-learning which simultaneously learns to represent the shapes of the dose profiles and to apply the most suitable regression functions—are the proposed solution. Such a solution will support several different experimental arrangements, offering optimum regression models for any such arrangement. By studying a few experimental cases, the effect of the selection of the experimental setup on the accuracy of parameter estimation has been demonstrated and discussed.

Estimation of the primary electron beam model parameters involves two steps, the first of which is an initial guess made by a regression model. In principle, this initial guess could be made without any such model—merely by a brute force search over all collected profiles for a set of profiles that best fit the analysed profiles. The second stage of estimation, which is based on reconstruction-based minimization, requires that techniques be developed to effectively represent the shape of the measured profiles—as introduced in the present work. It should also be noted that a brute force search delivers no explanatory power, in contrast to regression models introduced in the present study. In particular, regression models deliver an association between explanatory and explaining variables—for example, given a regression model it can be inferred in what manner will any specific changes of primary electron beam model parameters influence the shapes of the resulting dose profiles. This is the general advantage of regression models over any brute force search strategies, which is why regression models are widely used in statistical data analysis.

The developed framework was tested using both simulated and real data. The tests based on simulated data demonstrated that the coefficient of determination of true primary beam parameters from dose profiles varies from around 92% for angular beam divergence to 97% for mean energy of the simulated electron beam. It was not possible to train the developed model to predict the FWHM of energy spectrum of primary electrons, implying that this particular beam parameter does not seriously affect the shapes of dose profiles, at least for the cases studied in this work.

The presented framework has been made freely available together with the simulation data used for training the models. Model training and testing stages do not require extensive computation resources. Using any up-to-date PC with no graphic card support, the PCA + SVR models can be trained within a few seconds and prediction takes no longer than a second. The training of deep learning models usually requires about ten hours of an average CPU. However, testing the deep models takes no longer than testing the PCA + SVR model.

The presented framework can be readily adapted to individual requirements, perhaps guided by the availability of profile sets prepared for QA purposes, or by ease of measurement. Dose data could also be supplied by dose distributions measured by detectors other than ionization chambers, e.g., dye films, especially over regions of high dose gradient. Indeed, for any selection of profiles which the user intends to apply in determining values of parameters of the primary electron beam models, only a few lines of the configuration code need to be changed to indicate such user-specified selection. Then, the regression models must be retrained, which takes only a few seconds with no user intervention, except for running the code. Following this training run, the estimation of electron beam model parameters and reconstruction of profiles from estimation results can be executed—this requiring a few more seconds, provided that the measured doses are read by a script. Three examples of such procedures for reading measured doses from text files have also been provided in the freely available repository at https://github.com/taborzbislaw/DeepBeam.

## Conclusion

The purpose of the present study was to develop a flexible framework with suitable regression models for estimating parameters of the model of primary electron beam in simulators of medical linear accelerators, based on real reference dose profiles measured in a water phantom. The proposed framework is a readily applicable and customizable tool which may be applied in tuning parameters of primary electron beams of Monte Carlo simulators of linear accelerators. The codes, training and test data, together with readout procedures, are freely available at the site: https://github.com/taborzbislaw/DeepBeam.

## Supplementary Information

**Additional file 1.** Supplementary material.

**Additional file 2. Fig. S1.** Variance of profiles’ shapes explained by PCS. (a) Fraction of explained variance in the shapes of profiles, versus number of PCA features. The error bars represent standard deviation of the explained variance values, calculated for six profiles of three squared fields (3x3 cm2, 10x10 cm2 and 30x30 cm2); (b) Fraction of profile shape variance explained by the first three features, as averaged over the three squared fields, for six profiles (depth profile: ID=1, and five lateral profiles at depths Dmax=1.4 cm, 5 cm, 10 cm, 20 cm, and 30 cm, IDs from 2 to 6, respectively).

**Additional file 3. Fig. S2.** Variation in profiles’ shapes. Variation in profile shapes in relation to any one of the first three PCA features being either negative or positive (left, middle and right panels) for a 10x10 cm2, lateral profile at 30 cm depth (upper panels) or for a depth profile of a 10x10 cm2 field (lower panels). For explanation of “mean shape”, “negative feature” and “positive feature” labels, see text.

**Additional file 4. Fig. S3.** Selecting dose profiles. Coefficient of determination between ground truth and predicted values of energy, spot size, and angular divergence, for regression based on all of three fields (3x3 cm2, 10x10 cm2 and 30x30 cm2) and a different number of profiles of each field. For further details, see text.

## Data Availability

All data and code are available at https://github.com/taborzbislaw/DeepBeam.

## Abbreviations

MC: Monte Carlo
EBT: External beam photon therapy
TPS: Therapy planning system
QA: Quality assurance
FWHM: Full width at half maximum
PCA: Principal component analysis
SVR: Support vector regression
DL: Deep learning
