# Correction to: DeepBeam: a machine learning framework for tuning the primary electron beam of the PRIMO Monte Carlo software

**DOI:** 10.1186/s13014-022-01983-x

**Published:** 2022-02-28

**Authors:** Zbisław Tabor, Damian Kabat, Michael P. R. Waligórski

**Affiliations:** 1 AGH University of Science and Technology, Al. Adama Mickiewicza 30, 30-059 Kraków, Poland; 2 Maria Sklodowska-Curie National Research Institute of Oncology Krakow Branch, Garncarska 11, 31-115 Kraków, Poland; 3 Cracow University of Technology, Podchorążych 1, 30-084 Kraków, Poland

## Correction to: Radiat Oncol (2021) 16:124 https://doi.org/10.1186/s13014-021-01847-w

After publication of this article [1], the authors reported that the statement in the Funding information section was incorrectly given as “This study was supported by the Foundation for Polish Science under Grant POIR.04.04.00-00-15E5/18.” and should have read “This study was supported by the POIR.04.04.00-00-15E5/18 Project. The POIR.04.04.00-00-15E5/18 project is carried out within the TEAM-NET programme of the Foundation for Polish Science co-financed by the European Union under the European Regional Development Fund.”

The original article [1] has been updated.
