# Supplementary material for: DeepBeam: a machine learning framework for tuning the primary electron beam of the PRIMO Monte Carlo software
Source: Radiat Oncol. 2021 Jun 29;16:124. doi: 10.1186/s13014-021-01847-w (PMC8243564; doi:10.1186/s13014-021-01847-w)
Supplement: Supplementary file 1 — Additional file 1. Supplementary material. [file 13014_2021_1847_MOESM1_ESM.docx]

**Supplementary material**

**Related works**

In the study of Almberg et al. [9] an algorithm is proposed to determine values of two parameters (nominal energy and spot size) of the primary electron beam model, basing on depth and lateral photon beam profiles measured for three fields: 2x2, 10x10, and 20x20 cm2. The MC profiles are calculated for preselected values of nominal energies and spot sizes. The authors propose an ad hoc cost function, calculated from a range of hand-crafted features extracted from profiles, to select between some preselected nominal energy and spot size values those which minimize the cost function. The algorithm, iterating over preselected values, finds values of only two of the four model parameters. The tuning procedure proposed in the study of Pena et al. [12] is aimed at determining three parameters (the nominal energy, spot size, and angular divergence) of the primary electron beam model, basing on a depth and a penumbra profile for the 5x5 cm2 field and three lateral profiles for four fields: 5x5, 10x10, 20x20, and 40x40 cm2. The MC profiles are also calculated for preselected values of nominal energies and an arbitrarily fixed values of spot size and angular divergence. In the first step, the authors, using these fixed angular divergence and spot size values and applying a figure of merit, search for an optimal energy, iterating over MC results. Only the first step of this tuning procedure is formalized, because in the next step, after fixing the energy parameter to the value found in the previous step, an optimal spot size is searched for by trial and error, comparing measured and simulated penumbra for a 5x5 cm2 field. In the last step, the value of the angular divergence parameter is searched for, also by trial and error, using lateral profiles. The last two steps require an extensive MC simulation effort while being based on a trial-and-error approach. Other studies of [13-18] are also based on a trial-and-error approach which, while being computationally demanding, offers no systematic procedure for estimating parameter values in the applied model of the primary electron beam.

It follows that in the published papers describing tuning the model of the primary electron beam, ad hoc approaches are primarily used. In most cases a trial-and-error approach has been adopted. Other approaches rely on of searching for the beam model parameters using various figures of merit, over a predefined set of parameters for which MC results have been pre-computed. Within some hybrid approaches, the value of one of the model parameters is found by searching over a set of preselected values while values of the remaining parameters are found by trial and error - which again requires extensive MC simulation.

The published studies generally share common feature – they lack any phenomenological model enabling the values of primary electron beam parameters to be estimated directly from the measured dose profiles. They also suffer from specific, often quite demanding requirements with respect to experimental equipment and measurement procedures. As different measurement devices and different dosimetry procedures are typically used at various clinical centres, relaxing such constraints is highly desirable.

**Selecting the number of PCA features**

Results are shown in **Fig. S1a** separately for three analysed fields (3x3 cm^2^, 10x10 cm^2^ and 30x30 cm^2^) as averages over six profiles extracted from the simulated 3D dose distributions for each of these three fields (depth profile and five lateral profiles at depths D_max_ = 1.4 cm, 5 cm, 10 cm, 20 cm, and 30 cm). The error bars represent standard deviations of the values of explained variance, calculated over the six profiles of these three fields. Clearly, three PCA features suffice in explaining most of the variability of the shapes of profiles, so any further increase of PCA features above that number would not offer much in terms of added benefit.

Results shown in **Fig. S1b** illustrate the relative contributions that each of the three most important PCA features introduce in explaining the variance of profile shape. It is evident that the first PCA feature explains most of the variation in the depth profile (ID=1). The contributions offered by consecutive PCA features in explaining the overall variation in the shapes of lateral profiles increase with increasing depth at which these lateral profiles are extracted. It follows from this figure that use of lateral profiles extracted at larger depths offers more benefit in model predictions, as the variability of their shapes is richer (i.e., the contribution of higher PCA modes to the shape of the final profile is then more pronounced). Effectively, more information about the primary electron beam may be encoded in such profiles at larger depths.

The impact of the three major PCA features on the shape of dose profiles is shown in **Fig. S2** for two selected profiles (10x10 cm^2^ lateral profile at 30 cm depth - upper panels, and 10x10 cm^2^ depth profile – lower panels). Here, changes with respect to the mean profile shapes when varying any one of the three most important PCA features are shown. The PCA features of mean profiles are all equal to zero. Profiles marked in this figure as “Negative feature” have only the first, second, or third PCA feature negative in plots within the left, middle, or right panels, respectively. Similarly, profiles marked as “Positive feature” have only the first, second, or third PCA feature positive in respective left, middle, or right panels.

**Selecting dose profiles**

The coefficient of determination between ground truth and predicted values of energy, spot size, and angular divergence is shown in **Fig. S3**. The coefficient of determination was calculated for held-out parts of the full training set, in accordance with cross-validation approach. Results presented in this figure are obtained for regressors trained on different numbers of profiles of any of the three fields: 3x3 cm^2^, 10x10 cm^2^, and 30x30 cm^2^. For example, in the case of the number of profiles of each field being equal to one, only depth profiles of the three fields were used for regressor training. For the number of profiles of each field equal to two, depth profiles and lateral profiles at 1.4 cm depth of these three fields were used for regressor training. It follows from the results shown in this figure that a total of six profiles (one depth and one lateral), that is two from each of three (3x3 cm^2^, 10x10 cm^2^ and 30x30 cm^2^) fields, would suffice in precisely predicting the values of *E*, s, and α.

Note that in **Fig. S3** predictions of the FWHM of the primary beam energy distribution, σ*_E_*, have not been presented, as it was not possible to train a regressor to precisely predict the value of σ*_E_*. The effect of varying the value of σ*_E_* on the shapes of the profiles was noted to be very small – below 2%, in line with the noise level, which is the likely cause of lack of success in training regressors to predict the value of the parameter which represents the energy distribution of the primary beam. Thus, only *E*, s, and α (energy, spot size, and angular divergence) were further considered in the analysis.

The coefficient of determination between ground truth and predicted values of energy, spot size, and angular divergence for a limited selection of profiles (depth, lateral at depth of D_max_=1.4 cm and lateral at depth of 10 cm) and a varied number of fields for these profiles was also analysed. Application of a total of six profiles - one depth profile and two lateral profile, and any two of three fields (3x3 cm^2^, 10x10 cm^2^, or 30x30 cm^2^) would be sufficient in obtaining precise predictions of *E*, s, and α values. It appears however that three profiles extracted from a single field (except for the 3x3 cm^2^ field alone), would also be sufficient to precisely determine the values of primary electron beam parameters *E*, s, and α, with a coefficient of determination above 0.98.

**Supplementary material figure legends**

**Fig. S1** **Variance of profiles’ shapes explained by PCS.** (a) Fraction of explained variance in the shapes of profiles, versus number of PCA features. The error bars represent standard deviation of the explained variance values, calculated for six profiles of three squared fields (3x3 cm^2^, 10x10 cm^2^ and 30x30 cm^2^); (b) Fraction of profile shape variance explained by the first three features, as averaged over the three squared fields, for six profiles (depth profile: ID=1, and five lateral profiles at depths D_max_=1.4 cm, 5 cm, 10 cm, 20 cm, and 30 cm, IDs from 2 to 6, respectively).

**Fig. S2** **Variation in profiles’ shapes.** Variation in profile shapes in relation to any one of the first three PCA features being either negative or positive (left, middle and right panels) for a 10x10 cm^2^, lateral profile at 30 cm depth (upper panels) or for a depth profile of a 10x10 cm^2^ field (lower panels). For explanation of “mean shape”, “negative feature” and “positive feature” labels, see text.

**Fig. S3** **Selecting dose profiles.** Coefficient of determination between ground truth and predicted values of energy, spot size, and angular divergence, for regression based on all of three fields (3x3 cm^2^, 10x10 cm^2^ and 30x30 cm^2^) and a different number of profiles of each field. For further details, see text.
