# Supplementary figures and images for: DeepBeam: a machine learning framework for tuning the primary electron beam of the PRIMO Monte Carlo software
Source: Radiat Oncol. 2021 Jun 29;16:124. doi: 10.1186/s13014-021-01847-w (PMC8243564; doi:10.1186/s13014-021-01847-w)

(a)
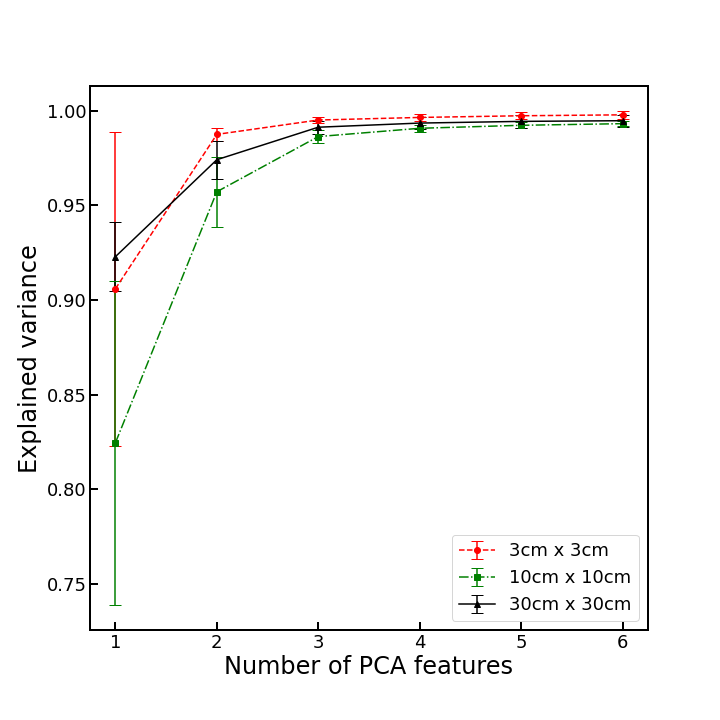
 (b)
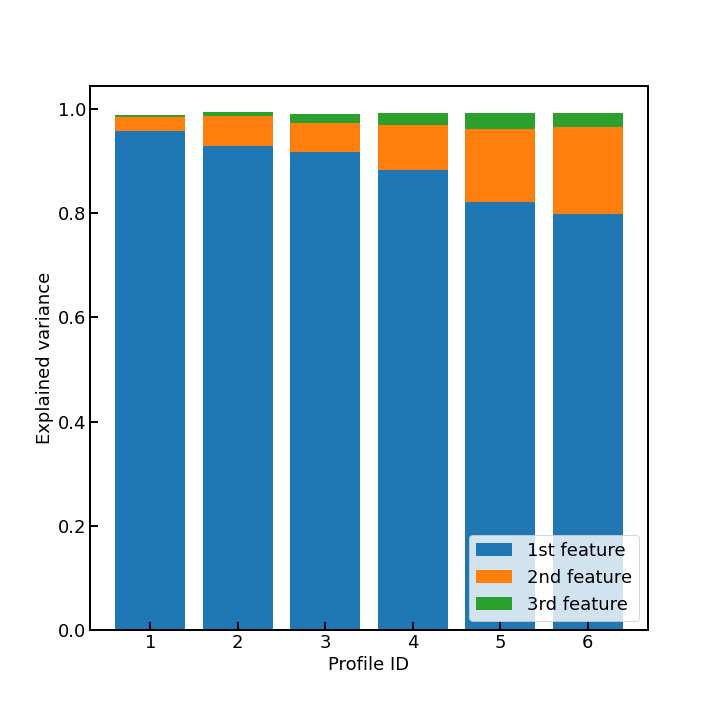

Supplement: Supplementary file 2 — Additional file 2. Fig. S1. Variance of profiles’ shapes explained by PCS. (a) Fraction of explained variance in the shapes of profiles, versus number of PCA features. The error bars represent standard deviation of the explained variance values, calculated for six profiles of three squared fields (3x3 cm2, 10x10 cm2 and 30x30 cm2); (b) Fraction of profile shape variance explained by the first three features, as averaged over the three squared fields, for six profiles (depth profile: ID=1, and five lateral profiles at depths Dmax=1.4 cm, 5 cm, 10 cm, 20 cm, and 30 cm, IDs from 2 to 6, respectively). [file 13014_2021_1847_MOESM2_ESM.docx]

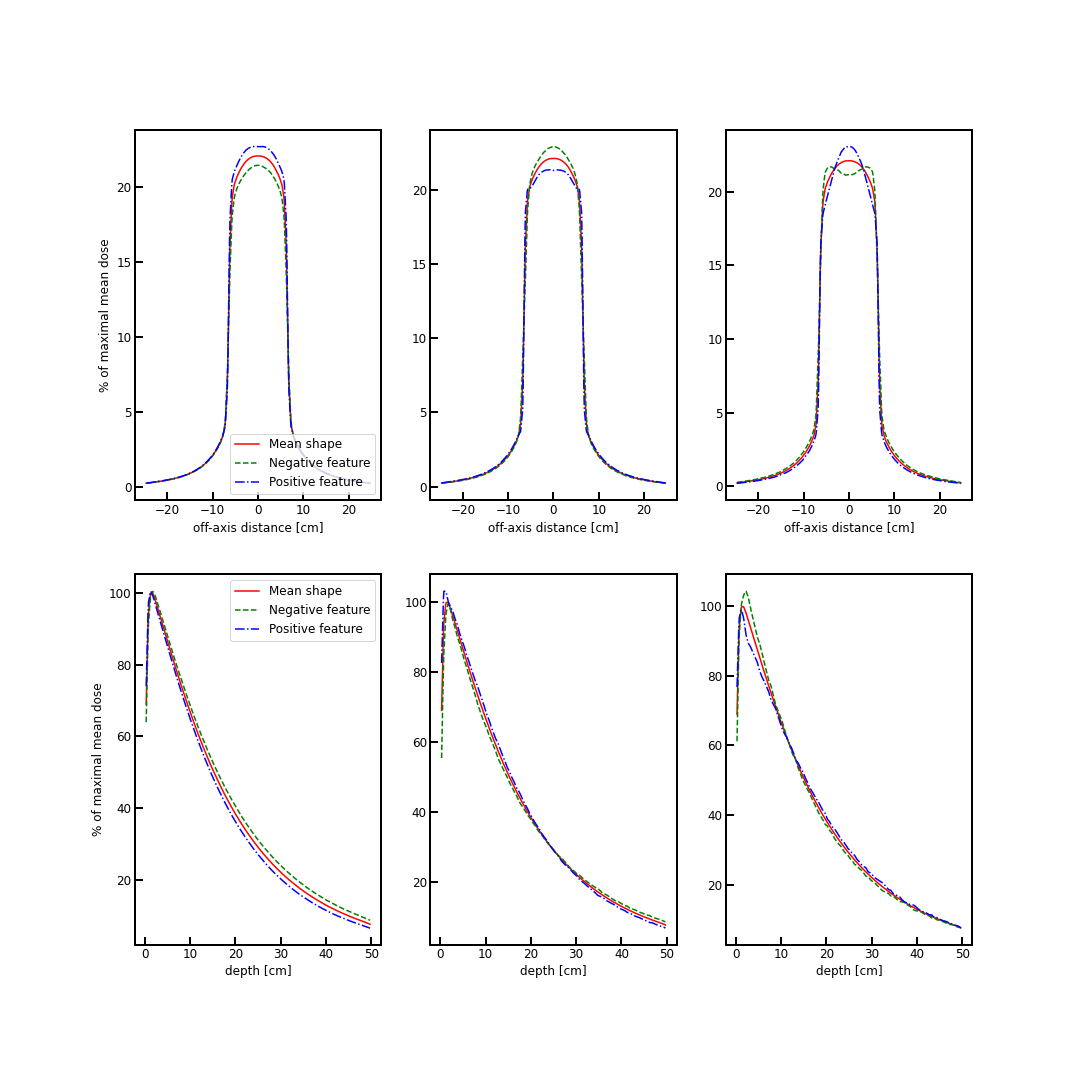

Supplement: Supplementary file 3 — Additional file 3. Fig. S2. Variation in profiles’ shapes. Variation in profile shapes in relation to any one of the first three PCA features being either negative or positive (left, middle and right panels) for a 10x10 cm2, lateral profile at 30 cm depth (upper panels) or for a depth profile of a 10x10 cm2 field (lower panels). For explanation of “mean shape”, “negative feature” and “positive feature” labels, see text. [file 13014_2021_1847_MOESM3_ESM.docx]

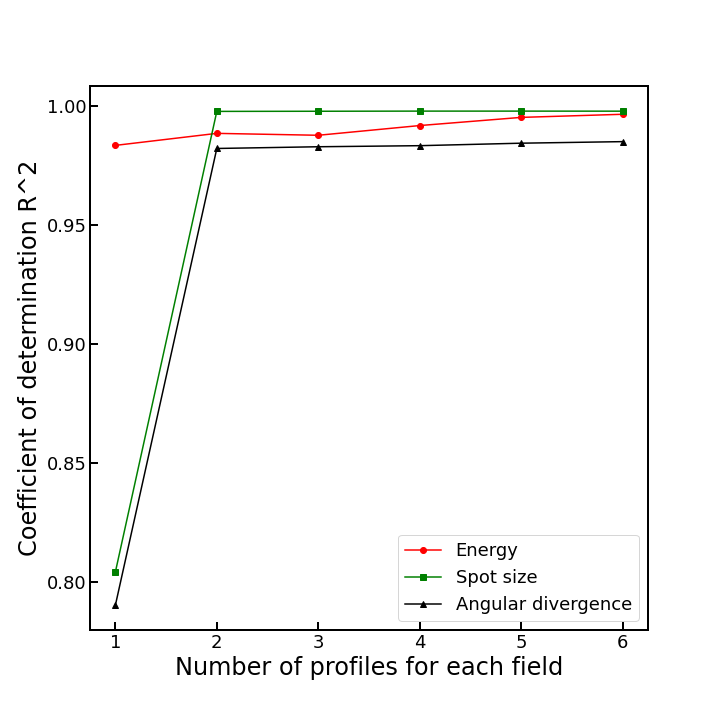

Supplement: Supplementary file 4 — Additional file 4. Fig. S3. Selecting dose profiles. Coefficient of determination between ground truth and predicted values of energy, spot size, and angular divergence, for regression based on all of three fields (3x3 cm2, 10x10 cm2 and 30x30 cm2) and a different number of profiles of each field. For further details, see text. [file 13014_2021_1847_MOESM4_ESM.docx]
